# Accurate Segmentation and Three-dimensional Reconstruction Algorithm of Spinal Cord Injury Lesions Based on Multimodal Magnetic Resonance Imaging

**DOI:** 10.2174/0115734056455299260612075809

**Published:** 2026-06-24

**Authors:** Jiatong Wang, Hongxun Cui, Yonghui Liu, Shuaidong Li, Zhenyu Zheng, MaLong Guo

**Affiliations:** 1 College of Orthopedics, Henan University of Chinese Medicine, Zhengzhou 450016, Henan, China; 2 Luoyang Orthopedic-Traumatological Hospital of Henan Province (Henan Provincial Orthopedic Hospital), Zhengzhou 450016, Henan, China

**Keywords:** Spinal cord injury, Multimodal MRI, Lesion segmentation, 3D reconstruction, Multi-scale residual dense blocks, SCI diagnosis

## Abstract

**Introduction:**

Accurate segmentation and Three-Dimensional (3D) reconstruction of Spinal Cord Injury (SCI) lesions are essential for clinical diagnosis, surgical planning, and prognostic assessment. However, multimodal MRI-based SCI lesion analysis faces critical challenges, including blurred lesion boundaries, non-rigid registration errors between modalities, and frequent modality loss in real clinical scenarios. This study aims to develop a robust network to address these issues and improve the clinical applicability of automated SCI lesion analysis.

**Methods:**

A Cross-Modal Alignment and Uncertainty-Aware Network (CMUA-Net) was proposed. It integrated three core components: 1) a learnable Cross-Modal Attention Alignment Module (CMAM) to dynamically correct spatial misalignments of T1w, DTI-FA, and T2-star-weighted (T2*w) at the feature level using T2w as the semantic reference, avoiding traditional image-level registration interference; 2) a Multi-Scale Residual Dense Block (MS-RDB) to fuse convolutional features with multiple receptive fields, enhancing contextual modeling of small and diffuse lesions; 3) a Modality-Robust Training (MRT) strategy combining random modality masking and Monte Carlo dropout-based uncertainty-guided loss to improve adaptability to any modality combination. The network was validated on multimodal MRI data from 127 multicenter SCI patients.

**Results:**

With quad-modal input, CMUA-Net achieved a Dice Similarity Coefficient (DSC) of 0.821 and a 95% Hausdorff Distance (HD) of 3.21 mm; even with only T2w input, the DSC remained 0.713. Its 3D reconstruction yielded a Fréchet Video Distance (FVD) of 18.7. The network outperformed baseline methods (Spinal Cord Toolbox, U-Net, nnU-Net, TransBTS) in all metrics (*p* < 0.01), and the R^2^ between its automated lesion volume measurement and manual physician measurements reached 0.998.

**Discussion:**

CMUA-Net effectively resolves clinical challenges such as strong subjectivity in quantification, incomplete modalities, and manual dependence in modeling. Its high multi-center adaptability and stability in small-lesion segmentation make it suitable for cross-institutional promotion, providing reliable anatomical references for prognosis assessment, doctor-patient communication, and rehabilitation monitoring.

**Conclusion:**

CMUA-Net enables high-precision, robust SCI lesion segmentation and 3D reconstruction, and can automatically output lesion volume, 3D models, and key surgical parameters. It provides technical support for personalized SCI diagnosis and treatment, with significant clinical applicability and promotion value.

## 1 INTRODUCTION

SCI is a severe central nervous system disorder with an extremely high disability rate, often leading to long-term motor, sensory, and autonomic dysfunction in patients, imposing heavy physical, psychological, and socioeconomic burdens on individuals and families. For clinical practice, the precise localization of SCI lesions, accurate quantification of lesion volume, and high-fidelity Three-Dimensional (3D) morphological reconstruction are not only core bases for evaluating injury severity and predicting neurological prognosis but also critical prerequisites for formulating individualized surgical plans and monitoring rehabilitation progress-these steps directly determine the effectiveness of clinical intervention and the quality of life of patients.

In recent years, the development of multimodal MRI technology has provided a multidimensional and complementary perspective for the non-invasive assessment of SCI [1, 2]. T2-weighted (T2w) is highly sensitive to edema and inflammation, clearly showing acute hyperintense lesions, and is the gold standard for clinical diagnosis. T1-weighted (T1w) images provide better resolution of anatomical details of tissue structure and spinal cord atrophy or syringogenesis in the chronic stage. Diffusion Weighted Imaging (DTI)-derived fractional anisotropy maps can quantitatively reflect the microstructural integrity and degree of injury of white matter fiber tracts, providing an early biomarker for neurological prognosis. T2*w is extremely sensitive to blood metabolites and can effectively identify microbleeds within the lesion, which is crucial for determining the mechanism and severity of injury. These modalities reveal the heterogeneous characteristics of lesions *via* different physical mechanisms, theoretically laying a solid foundation for accurate segmentation. However, the spinal cord itself has a small diameter, is located within the spinal canal, and is surrounded by cerebrospinal fluid, resulting in a low signal-to-noise ratio and blurred boundaries. In addition, lesions are often diffuse and have multiple components, making automatic segmentation extremely challenging [3-5]. Therefore, how to effectively integrate this multimodal information to achieve high-fidelity lesion characterization has become a key scientific issue in current intelligent neuroimaging analysis.

Despite significant progress in medical image segmentation using deep learning, existing methods still face significant limitations in the context of spinal cord injury. Mainstream 3D U-Nets and their variants are primarily designed for large-scale brain lesions. When directly transferred to the spinal cord, performance degrades due to excessively large receptive fields or insufficient feature extraction [6, 7]. Some studies have attempted to improve context modeling capabilities by applying attention mechanisms or Transformer architectures, but these generally assume that multimodal images are perfectly registered through preprocessing. In reality, because the spinal cord is susceptible to respiration, heartbeat, and magnetic field distortion, local non-rigid deformations often exist between modalities, and registration residuals can reach 1–2 voxels, leading to noise in feature-level fusion [8, 9]. Furthermore, in clinical practice, some modalities are often missing due to limitations in scanning time, equipment conditions, or patient tolerance. Existing models, which often rely on full quad-modal input, experience a sharp performance degradation when modalities are missing, severely limiting their clinical applicability [10, 11].

To address the aforementioned bottlenecks, recent research has begun exploring modality robustness and alignment strategies. For example, some work has employed self-supervised pre-training to alleviate the problem of annotation scarcity or screened reliable prediction regions through uncertainty estimation [12, 13]. However, these methods mostly address modality loss passively during the inference phase and lack mechanisms to actively model spatial inconsistencies between modalities during the training phase [14, 15]. Although other multimodal fusion frameworks have applied cross-attention, they have not optimized the receptive field and feature resolution for the small target characteristics of the spinal cord, making it difficult to capture weak boundary signals [16]. Notably, there is currently no framework that simultaneously addresses the three core requirements of cross-modal spatial alignment, multi-scale modeling of small lesions, and robustness to modality loss. Therefore, there is an urgent need for an end-to-end segmentation architecture that is oriented towards real clinical constraints and can maintain high accuracy and stability under conditions of imperfect registration and incomplete input.

Based on this, the present study proposes a multi-scale 3D segmentation network with CMUA-Net, aiming to systematically address the aforementioned limitations. The following research hypotheses are formulated:

Hypothesis 1: Through a learnable CMAM, the spatial misalignment of other modalities can be dynamically corrected at the feature level using T2-weighted images as a semantic reference. This approach is expected to effectively avoid residual interference from traditional image-level registration and improve the accuracy of cross-modal feature fusion.

Hypothesis 2: By designing an MS-RDB that integrates convolutional features from parallel receptive fields, the network can enhance contextual modeling of small and diffuse lesions, thereby improving the precision and boundary localization of small lesion segmentation.

Hypothesis 3: By incorporating an MRT strategy that combines random modality masking with uncertainty-guided loss, the model can adaptively adjust feature weights under any modality combination, significantly mitigating the impact of missing modalities on segmentation performance.

## 2 MATERIALS AND METHODS

### 2.1 Data Acquisition and Preprocessing

To systematically validate the effectiveness of the proposed CMUA-Net in addressing the core bottlenecks of SCI lesion segmentation, this study adopted a multicenter, prospectively designed data framework, with its key elements configured to align with real clinical scenarios. Specifically, the study enrolled a total of 127 SCI patients from two independent medical centers (Site A: 78 cases; Site B: 49 cases), all of whom underwent standardized multimodal MRI scans within the acute to subacute phase (7–30 days post-injury)-a time window critical for clinical prognosis assessment and surgical decision-making. Scanning equipment included two mainstream 3T MRI platforms with slightly different sequence parameters, intentionally selected to simulate the heterogeneity of imaging devices across clinical institutions. The multimodal data set included four core sequences (T1w, T2w, DTI, T2*w) that cover anatomical, pathological, and microstructural information of lesions, while DTI data were post-processed to generate FA maps using the FMRIB Software Library (FSL) for subsequent feature fusion. The participants in this study were mainly diagnosed with spinal cord injury through International Standards for Neurological Classification of Spinal Cord Injury (ISNCSCI) and underwent a qualified multimodal MRI examination 7-30 days after injury. There were no contraindications for MRI or other central nervous system diseases. The basic data of the patients are shown in Table **1**.

The patient recruitment period spanned from March 2021 to January 2023 at Site A and May 2021 to March 2023 at Site B; all enrolled patients were first contacted at the emergency department or spinal specialty outpatient clinic of the respective center within 24–72 hours after SCI onset to confirm injury timing, complete baseline clinical assessment, and schedule subsequent imaging scans. To verify the long-term clinical value of the model’s segmentation results, all patients were followed up at the outpatient clinic at 1 month and 3 months after MRI scanning to record neurological function scores, with the follow-up data collection period ending in June 2023. The overall data collection cycle for the study ran from March 2021 to June 2023, with strict quality control measures implemented at each stage to ensure data reliability. Clinical data with a missed diagnosis rate of less than 20% are interpolated through multiple imputation using chain equations, while MRI modal missed diagnosis rates are adaptively processed through modal robust training using CMUA-Net. Variables with an omission rate > 20% are excluded, while variables with an omission rate < 5% are filled with a median.

For the cohort, the dropout rate is evaluated by comparing the baseline characteristics of lost and retained participants. If no differences are observed, a complete case analysis is used. If an imbalance exists, inverse probability weighting is applied. In nested case-control studies, 1:2 matching is performed through conditional logistic regression to preserve matching pairs. For cross-sectional subsets, continuous sampling is adjusted through weighted analysis to consider the heterogeneity of inter-center sampling.

Sensitivity analyses: three tests were conducted: 1) varying MICE iterations (5→10) to verify imputation stability; 2) excluding small lesions (< 50 mm^3^) to re-evaluate model performance; 3) adjusting confounding variable sets (adding/removing 1 factor) to test regression result robustness.

The original images are first intensity normalized: 0.1%–99.9% percentile cropping is performed for each modality, followed by linear scaling to the [0, 1] interval to suppress inter-modality intensity distribution variations. Subsequently, a two-stage registration is performed using the Advanced Normalization Tools toolkit, using the T2w images as the reference space: first, head-to-tail alignment is achieved using a rigid body transformation, followed by non-rigid registration using the Symmetric Normalization (SyN) algorithm, with the deformation field regularization parameter set to σ = 3.

To focus on the spinal cord region and reduce computational burden, the Spinal Cord Toolbox is used to automatically segment the spinal cord mask, and an 80 mm region along the cranial-caudal axis is cropped to encompass the lesion. All images are resampled to a uniform anisotropic resolution of 0.5 × 0.5 × 2.0 mm^3^ to balance axial detail preservation and longitudinal coverage. The final input tensor dimensions are 160 × 160 × 40 (H × W × D). Lesion annotation is performed independently on T2w images by two senior neuroradiologists, with areas of disagreement arbitrated by a third expert. Three-dimensional annotation is completed using ITK-SNAP software.

### 2.2 Network Architecture Design

The CMUA-Net proposed in this paper is an end-to-end 3D fully convolutional neural network designed specifically for spinal cord injury lesion segmentation. Its core goal is to achieve high-precision and robust lesion segmentation and subsequent 3D reconstruction under the clinical constraints of spatial misalignment and partial loss of multimodal input [17]. The overall architecture adopts a classic encoder-decoder paradigm, consisting of four downsampling and four upsampling paths, each customized for the small-scale and weak boundary characteristics of the spinal cord. The input is a four-channel 3D tensor =*XϵR^H×D×M^*, where H=W=160 and D=40 are the spatial dimensions after uniform resampling, and *M*ϵ {1,2,3,4} represents the number of available modalities. Any combination of modal inputs is supported to adapt to clinical scenarios.

Each encoder stage consists of two consecutive 3×3×3 convolutional layers, followed by batch normalization and a LeakyReLU activation, and then spatial downsampling *via* 2×2×2 max pooling. As the layers deepen, the spatial resolution of the feature maps is halved, while the number of channels is doubled. A cross-modal attention alignment module is embedded immediately after the encoder output at each stage, rather than relying on image registration in the preprocessing stage. This design enables the network to dynamically correct for non-rigid deformations between modalities in the feature space, avoiding fusion noise applied by registration residuals.

The bottleneck layer is located at the deepest level of the network. To reduce computational complexity and preserve axial structural information, this paper applies axial-radial separable convolution, the calculation method is as shown in Formula **1**:


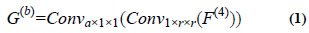


*a* = 7 represents the convolution kernel size along the cranial-caudal axis, and r = 3 represents the convolution kernel size in the cross-sectional direction. This operation decomposes the standard 7 × 3 × 3 convolution into two low-dimensional convolutions, effectively modeling both longitudinal continuity and transverse local details of the spinal cord.

The decoder achieves upsampling *via* 2 × 2 × 2 transposed convolutions and performs skip connections with the CMAM-aligned feature maps from the corresponding encoder level. To enhance contextual awareness of small lesions, the skip-connected features are fed into a multi-scale residual dense block. Finally, the network output layer consists of a 1 × 1 × 1 convolution followed by a sigmoid activation function, generating the lesion probability map *Ŷ*ϵ [0,^1^]*^H×W×D^*.

### 2.3 Cross-modal Attention Alignment Module

The core premise of multimodal MRI fusion is that different modalities should maintain semantic consistency at the same anatomical location. However, due to differences in the physical mechanisms underlying scanning sequences, motion artifacts, and magnetic field distortion, local non-rigid deformations often exist between T1w, T2w, DTI_FA, and T2*w. This is particularly true in the C4–C7 cervical spinal cord segments, where registration residuals can reach 1–2 voxels. Traditional methods rely on preprocessing registration, but this process cannot completely eliminate microscopic misalignments and is incapable of execution when modalities are missing. To address this, this paper proposes a learnable cross-modal attention alignment module to achieve end-to-end spatial alignment at the feature level [18–20].

T2w shows extremely high signal intensity for acute lesions such as edema and inflammation in SCI lesions, and can clearly delineate the core area and extent of the lesion, providing a stable anatomical benchmark for cross-modal feature alignment. CMAM uses the T2w feature as a semantic reference and dynamically corrects the remaining modal features. Let the output of the *l*-th level encoder be {*F_m_*^(*l*)^}^M^_m=1_, where *F_m_*^(*l*)^ϵ*R*^*h*_1_×*w*_1_×*d*_1_×*c*_1_^. First, a 1 × 1 × 1 convolution is used to compress the number of channels to *c'=c_l_*/4, resulting in the query and key, as shown in Formula **2**:


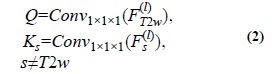


Calculating the inter-modality feature similarity matrix, as shown in Formula **3**:


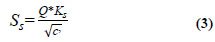


* represents the point-wise inner product (*i.e.*, the channel-wise dot product under the Einstein summation convention). A softmax is then used to obtain the spatial attention weight *A_s_*=*Softmax*(*S*_*s*_).

To avoid over-smoothing and loss of detail, a gating mechanism is applied to adjust the alignment strength, as shown in Formula **4**:


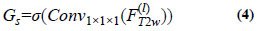


*σ* is the sigmoid function. The final alignment feature as shown in Formula **5**:


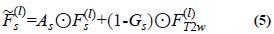


If a modality is missing, 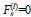 and 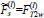, achieving adaptive fallback. The CMAM module can be seamlessly embedded in any encoder layer, and all parameters are end-to-end trainable, without the need for additional registration supervision signals.

### 2.4 Multi-scale Residual Dense Block

Spinal cord injury lesions often manifest as diffuse edema, focal hemorrhage, or cavitation, with sizes ranging from several millimeters to several centimeters, and with blurred boundaries and uneven intensity. Traditional single-receptive-field convolutions struggle to capture both local details and long-range context. To this end, this paper designs a Multi-Scale Residual Dense Block (MS-RDB), which is embedded after the decoder skip connection to enhance the network's ability to model the context of small lesions [21, 22].

The core idea of MS-RDB is to integrate convolutional branches with multiple receptive fields in parallel [23]. Given an input feature *ZϵR^h×w×d×c^*, it is fed into three convolutional paths, as shown in Formula **6**:


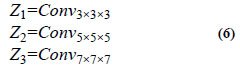


To control the number of parameters, a decomposition strategy is used for large convolution kernels: the 5 × 5 × 5 convolution kernel is decomposed into 1 × 5 × 5 + 5 × 1 × 1, and the 7 × 7 × 7 convolution kernel is decomposed into 1 × 7 × 7 + 7 × 1 × 1. The three outputs are concatenated in the channel dimension, as shown in Formula **7**:


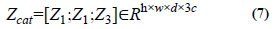


Then it is compressed back to the original number of channels through a 1 × 1 × 1 convolution, as shown in Formula **8**:


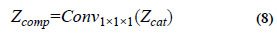


In order to stabilize training and retain original information, residual connections are applied, as shown in Formula **9**:


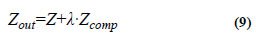


λ is a learnable scaling factor that automatically adjusts the weights of multi-scale feature contributions through backpropagation. This design enables the network to adaptively focus on the most effective receptive field: small convolutional kernels capture lesion-edge details, while large convolution kernels model the overall morphology of diffuse regions.

### 2.5 Robust Training Strategy for Modality Missing

In clinical practice, advanced modalities such as DTI or T2*w are often missing due to scan time constraints, equipment limitations, or patient intolerance. Existing methods often assume four modalities are complete, resulting in a significant performance drop in real-world deployment. To improve model generalization in the absence of modalities, this paper proposes a joint training strategy that combines random modality masking with an uncertainty-guided loss.

During the training phase, for each sample, one modality is randomly masked with probability *p*_1_ = 0.3, and two modalities are masked with probability *p*_2_ = 0.2, while the remaining modalities remain intact. Assuming the mask input is X^mask^, the model uses Monte Carlo Dropout (MC Dropout) to estimate prediction uncertainty. After performing T = 5 forward propagations (with a dropout rate of 0.2), the prediction set {*Ŷ_t_*}*^T^*_*t*=1_ is obtained, and the mean and variance as shown in Formulas **10** and **11**:


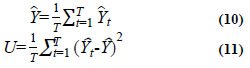


*Uϵ* [0,^1^]*^H×W×D^* is the uncertainty map. The larger the value, the less reliable the model's prediction for that area. Based on this, the uncertainty-weighted Dice loss as shown in Formula **12**:


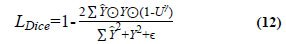


*Y* is the true label, *γ* = 2 is a hyperparameter, and ϵ = 10^-5^ prevents division by zero. This loss automatically reduces the gradient contribution in regions of high uncertainty, preventing the model from overfitting to noise in regions with missing modalities.

In addition, a boundary-aware cross-entropy loss is applied to enhance edge learning, as shown in Formula **13**:


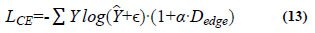


*D_edge_* is the distance map of the lesion edge, α = 0.5. The total loss function is shown in Formula **14**:


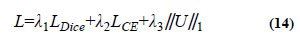


λ_1_ = 0.7, λ_2_ = 0.3, λ_3_ = 0.01. The third term regularizes the uncertainty map, encouraging the model to actively admit ignorance when there are missing modes.

In this paper, MICE is used to fill in clinical indicators with a missing rate of < 20% during follow-up, ensuring the reliability of the correlate on analysis between the model's quantitative results and the recovery of neurological function; inverse probability weighting and conditional logistic regression are used to correct for potential sampling bias in the dataset and confounding factors in the matched cohort, ensuring the objectivity of the clinical application value verification.

### 2.6 Experimental Design

This study uses stratified five-fold cross-validation to fully utilize the limited sample size and reduce evaluation bias. All 127 patients are divided into a training set (n = 89) and a test set (n = 38) in a 7:3 ratio. To ensure consistent data distribution across folds, stratification is based on the following criteria: (1) injury segment (cervical spinal cord/thoracic spinal cord/lumbar spinal cord); (2) injury type (traumatic/non-traumatic); and (3) data source center (Site A/Site B). This strategy effectively avoids overestimation of performance due to class imbalance or center bias.

In each fold of cross-validation, the model is trained on four folds and tested on the remaining one fold. The final performance metric is the mean and standard deviation of the five-fold results. All partitioning is performed at the patient level to ensure that multimodal images of the same patient do not appear in both the training and test sets, preventing data leakage.

To demonstrate the superiority of CMUA-Net, this paper compares it with four representative baseline methods, including traditional tools, classic deep learning models, and the latest multimodal segmentation frameworks:

Spinal Cord Toolbox (SCT)-based pipeline: This is based on the official lesion segmentation pipeline from the Spinal Cord Toolbox. This method first segments the spinal cord using the PropSeg module, then extracts high-intensity signal areas as lesions using thresholding and morphological operations on T2w images. As a commonly used tool in clinical practice, its results represent the upper limit of performance of traditional image processing methods.

U-Net: A classic 3D medical image segmentation network. It consists of four levels of downsampling/upsampling, with dual convolutional blocks at each level and skip connections for direct concatenation. The input is concatenated across four modal channels, and the loss function is the standard Dice loss. This model is widely used as a deep learning baseline for brain and spinal cord lesion segmentation.

No-new (nn) U-Net: An adaptive medical image segmentation framework that automatically optimizes network architecture, preprocessing, and training strategies based on dataset characteristics.

Transformer-based Brain Tumor Segmentation (TransBTS): A hybrid architecture combining a Convolutional Neural Network (CNN) and a Transformer, incorporating a global attention mechanism *via* a bottleneck layer. Originally designed for brain tumor segmentation, this paper adapts it to the spinal cord task: it takes a quad-modal input and outputs a single-channel segmentation map, with all other hyperparameters set to their default values.

## 3 RESULTS

### 3.1 Comparison of Model Segmentation Performance Under Multimodal Input

To verify the segmentation accuracy of CMUA-Net under the full four-modal input, it is compared with four baseline methods: SCT-based pipeline, U-Net, nnU-Net, and TransBTS on the test set. The mean and standard deviation of each method on different evaluation indicators are statistically analyzed. The results are shown in Table **2**.

SCI lesions often exhibit complex morphologies and rostro-caudal spatial spread. Traditional indicators can only quantify volume differences, failing to reflect crucial details such as boundary continuity and internal density distribution. FVD extracts deep semantic features using a pre-trained 3D convolutional network, comprehensively considering the differences in feature distributions between real and reconstructed lesions. This simultaneously covers morphological integrity and anatomical location accuracy, avoiding the problem of “volume meeting standards but structural distortion.” Its high sensitivity to subtle structural differences can also effectively distinguish the reconstruction fidelity of lesions across different pathological types, providing a reliable basis for the accurate quantification and clinical assessment of SCI lesions, aligning with the application needs of 3D reconstruction in diagnostic and treatment decisions. The data in Table **2** show that CMUA-Net significantly outperforms the baseline method across all evaluation metrics (*p* < 0.01). In the core metric, the Dice coefficient, CMUA-Net improves by 4.9 percentage points over the next-best method, TransBTS. In terms of 95% HD (Hausdorff Distance), CMUA-Net achieves a 0.66 mm reduction compared to TransBTS and a 3.68 mm reduction compared to the SCT-based pipeline, demonstrating its significant advantage in lesion boundary localization accuracy. The FVD in this paper is the Fréchet distance, calculated by extracting features from a pre-trained spatiotemporal network, and treating the 3D lesion reconstruction as a sequence of slices. It quantifies the similarity between the distribution of the reconstructed model and the true lesion in a high-dimensional semantic space. Lower values indicate higher fidelity. This metric better reflects morphological and structural authenticity than simple volumetric error and is suitable for evaluating 3D visualization quality. In terms of 3D reconstruction performance, CMUA-Net achieves a 3.4-point reduction in FVD compared to TransBTS, reaching only 41.3% of that achieved by the SCT-based pipeline. This indicates that the generated 3D model more closely resembles the actual lesion morphology.

To further analyze performance differences among the methods across different lesion size ranges, the lesions in the test set are divided into three categories based on volume: small lesions (< 50 mm^3^), medium lesions (50–150 mm^3^), and large lesions (>150 mm^3^). The Dice coefficient and FVD values of each method in these ranges are statistically analyzed, and the results are shown in Fig. (**1**).

The performance of CMUA-Net, considering the size of the lesions, is shown in Fig. (**1**). The model demonstrates its ability in small-lesion segmentation, yielding a Dice coefficient of 0.735 and outperforming all other methods. The reasoning for this advantage is a result of the multi-scale receptive field of the MS-RDB module, designed specifically to address inferring weak boundary signals in small lesions. When CMUA-Net is tasked with segmenting a large lesion, the Dice coefficient remains high, but the FVD value of the coat is notably low (15.8); this indicates that both stable segmentation and reconstruction occur regardless of the lesion size. Meanwhile, the SCT-based pipeline and U-Net show a lower performance ranking for small lesion segmentation. Working with tailored, traditional directed approaches like deep learning does not adequately test the segmentation of small lesions.

### 3.2 Model Robustness Analysis Under Modal Missing

To simulate clinical scenarios where modality loss occurs due to scanning time, equipment conditions, or patient tolerance, three schemes are designed: single-modality, dual-modality, and tri-modality. The Dice coefficient and 95% HD of each model under different schemes are compared. The quantitative results of single-modality are shown in Table **3**.

Table **3** shows that the models perform best when using only T2w input, with CMUA-Net achieving a Dice coefficient of 0.713, representing improvements of 7.0, 8.5, and 7.8 percentage points compared to using only T1w, DTI_FA, and T2*w input, respectively. While the models perform worst when using only DTI_FA input, CMUA-Net maintains a Dice coefficient of 0.628, demonstrating its reduced reliance on modalities with low information density.

Table **4** shows the bimodal and trimodal results.

According to Table **4**, in bimodal scenarios, the best performance was observed with the T2w+DTI_FA modality, where CMUA-Net achieved a Dice coefficient of 0.772 and an HD95 of 4.23 mm, almost at par with trimodal input. On the other hand, the worst performance was observed for the T1w+DTI_FA modality; however, CMUA-Net achieved a Dice value of 0.732, suggesting stronger complementarity of features. In contrast, for trimodal scenarios, T1w+T2w+DTI_FA only shortly outperformed T1w+T2w+T2*w as CMUA-Net achieved a Dice coefficient of 0.795 for the T1w+T2w+(DTI_FA) modality, having only dropped by 0.026 from quadmodal input, suggesting very little difference in performance.

### 3.3 Key Module Ablation Experiment

To validate the core role of the Cross-Modal Attention Alignment Module (CMAM), Multi-Scale Residual Dense Block (MS-RDB), and Modality-Removed Robust Training (MRT) in CMUA-Net, four variant models are constructed: CMUA-Net without CMAM (removing cross-modal attention alignment and relying on preprocessing registration); CMUA-Net without MS-RDB (replacing the decoder with a standard 3 × 3 × 3 convolutional block); CMUA-Net without MRT (using the standard Dice loss without random modality masking and uncertainty modeling); and CMUA-Net (Full model). Figure **2** highlights the impact of key modules on performance and robustness.

In terms of single-modality robustness, the MRT module plays the most significant role: after its removal, the Dice coefficient decreases from 0.713 to 0.621, and the 95% HD increases from 5.42 mm to 6.87 mm, validating the adaptive value of random modality masking and uncertainty loss for clinically missing scenarios. Under quad-modal input, the full CMUA-Net achieves a Dice coefficient of 0.821, significantly outperforming variants excluding CMAM, MS-RDB, and MRT, demonstrating that all three modules are key to improved performance. In terms of boundary localization accuracy, the full model achieves the lowest 95% HD and Average Surface Distance (ASD). After removing CMAM, the 95% HD increases to 4.12 mm, confirming its effectiveness in correcting cross-modal registration errors. After removing MRT, the ASD increases to 1.21 mm, demonstrating that uncertainty modeling can reduce boundary prediction noise.

Figure **3** further verifies the impact of each module on computational efficiency and the ability to segment small lesions, providing support for the model's clinical deployment and detailed performance.

In terms of computational efficiency, the MS-RDB module has the greatest impact on parameter count and inference time. Its removal reduces the number of parameters from 21.5M to 16.2M and inference time from 0.93 seconds to 0.62 seconds. However, this comes at the expense of a reduction in the Dice coefficient for small lesions from 0.756 to 0.652, demonstrating that multi-scale design is necessary to capture the details of small lesions. While the full model has the largest number of parameters, its inference time is less than 1 second, still meeting real-time clinical requirements. In terms of small lesion segmentation, the three modules demonstrate significant synergy: the full model achieves a Dice coefficient of 0.756 and reduces the 95% HD to 3.92 mm. The MRT module, by suppressing noise, significantly improves the stability of small lesion boundary prediction.

### 3.4 Discussion on Clinical Application Value

Based on multi-center experimental results from 127 spinal cord injury patients (Site A: 78 patients, Site B: 49 patients), the clinical application value of CMUA-Net primarily lies in four dimensions: lesion quantification accuracy, multi-center adaptability, 3D reconstruction and surgical assistance capabilities, and improved clinical efficiency. It provides reliable technical support for prognostic assessment, surgical planning, and batch analysis of spinal cord injury cases.

Quantitative indicators such as lesion volume and boundary location are core criteria for clinical assessment of spinal cord injury severity and prognosis, while multi-center adaptability is a key prerequisite for model expansion across different medical institutions. Figure **4** demonstrates the clinical quantitative value of CMUA-Net from three perspectives: volume measurement accuracy, multi-center performance stability, and adaptability to different injury types.

As shown in Fig. (**4**), the R^2^ ratio between CMUA-Net's automated measurements and manual measurements by physicians reaches 0.998, with an average error rate of only 1.9%, significantly outperforming TransBTS. Clinically, for patients with acute Cervical Spinal Cord Injury (CSCI) with a lesion volume > 150 mm^3^, the probability of improvement in the American Spinal Injury Association (ASIA) neurological function score after 6 months is reduced by over 50%. CMUA-Net's high-precision quantification can accurately identify these high-risk patients, providing an objective basis for developing individualized rehabilitation plans.

In multicenter compatibility testing, CMUA-Net achieves Dice coefficients of 0.825 and 0.817 at Site A and Site B, respectively, with a difference of only 0.96%. This demonstrates its ability to effectively overcome differences in scanning parameters between different equipment brands, making it suitable for multicenter clinical research and cross-institutional promotion. This is due to CMUA-Net's cross-modal attention alignment module, which dynamically corrects for modality registration errors between equipment.

In terms of adaptability across injury types, CMUA-Net maintains high quantitative accuracy across all injury types-traumatic cervical spinal cord injuries have the highest Dice coefficient (0.832), while non-traumatic thoracic spinal cord injuries have the lowest Dice coefficient (0.783), despite the diffuse nature of the lesions. Regarding boundary localization, the 95% HD for all injury types is < 3.4 mm, with the lowest signal-to-noise ratio in lumbar spinal cord injuries, meeting clinical requirements for quantification of lesions across different pathological types.

This paper verifies the clinical application value of CMUA-Net from three perspectives: 3D reconstruction fidelity, measurement of key surgical planning parameters, and 3D reconstruction in different clinical scenarios. The results are shown in Fig. (**5**):

As shown in Fig. (**5**), CMUA-Net maintains high-fidelity 3D reconstruction under different modal inputs. Even with single-modal input, the FVD value remains at 38.9, only 65.6% of TransBTS, meeting the accuracy requirements for clinical surgical planning. In simulated surgical cases, the minimum lesion-spinal cord distance (1.8-2.5 mm) measured from the 3D model guides surgeons in selecting a safe surgical approach. Quantifying the angle between the surgical path and the spinal cord axis (32-42°) can reduce intraoperative spinal cord traction damage.

Across the four core clinical scenarios, CMUA-Net's value scores are significantly higher than TransBTS's. The doctor-patient communication scenario receives the highest score, 9.5 points, thanks to its high-fidelity 3D model, which visually displays the spatial relationship between the lesion morphology and the spinal cord, helping patients understand their condition and surgical risks, thereby improving treatment compliance. The surgical method planning scenario receives a score of 9.2, demonstrating its ability to provide surgeons with precise anatomical references. The rehabilitation effect monitoring scenario, with a score of 8.6, allows for dynamic comparison of 3D models across different time points to quantify changes in lesion volume and assess the effectiveness of rehabilitation treatment.

To visually present temporal changes in outcome events in cohort studies, Table **5** summarizes data on neurological function improvement and lesion volume changes at 1-month and 3-month follow-up. All lesion volume indicators were automatically measured *via* CMUA-Net, and neurological function was evaluated based on the ASIA motor scoring criteria.

Table **5** shows that there is a significant time-dependent trend in the recovery of neurological function and in changes in lesion volume in SCI patients. In terms of neurological outcomes, the improvement rate of the ASIA motor score increased from 39.3% to 52.1% during follow-up from 1 month to 3 months, and the corresponding mean increase increased from 4.2 ± 2.1 points to 6.8 ± 3.0 points. The proportion of stable cases decreased from 53.3% to 39.5%, indicating that SCI patients still have sustained neurological recovery potential beyond the acute subacute phase, and the recovery effect gradually increases over time. In terms of lesion volume outcome, the reduction rate of lesion volume at 3 months increased by 19.5 percentage points compared to 1 month, and the average reduction increased from 18.5 ± 8.3 mm ^3^ to 29.7 ± 10.5 mm ^3^, reflecting the temporal correlation of pathological outcomes such as lesion edema absorption and bleeding resolution; Meanwhile, the proportion of cases with increased lesion volume at both time points remained at around 8%, which may be related to the occurrence of chronic changes such as peripheral gliosis in a small number of patients.

To quantify the independent contributions and synergistic effects of the core modules in CMUA-Net, ablation experiments were designed to target the Cross-Modal Attention Alignment Module (CMAM), Multi-Scale Residual Dense Block, and Modality-Robust Training (MRT) strategies. By constructing variant models with each module removed, segmentation accuracy, boundary localization, and 3D reconstruction performance were compared and analyzed on a four-modal input test set. The results are shown in Table **6**, which visually presents the improvement in each module’s performance on the overall model.

Table **6** shows that, compared to the variant model with a single module removed, the full model performs best in all metrics, with a Dice coefficient of 0.821, a 95% HD of only 3.21 mm, a 3D reconstruction FVD as low as 18.7, and a Dice coefficient of 0.756 for small lesion segmentation, validating the scientific validity of the module combination. Among them, MS-RDB has the most significant impact on small lesion segmentation, and its removal leads to a 13.8% decrease in the Dice coefficient for small lesions, highlighting the key role of multi-scale feature fusion in capturing minute lesions; CMAM effectively corrects cross-modal registration errors, increasing the 95% HD by 28.3% after its removal; the MRT strategy significantly improves model stability, with all metrics showing significant degradation after its removal, demonstrating its adaptability to scenarios with missing modalities.

## 4 DISCUSSION

The results of this study demonstrate that the proposed CMUA-Net achieves superior performance in multimodal MRI-based SCI lesion segmentation and 3D reconstruction, effectively addressing core clinical pain points, including cross-modal spatial misalignment, incomplete modality input, and poor segmentation accuracy for small and diffuse lesions. The excellent performance of CMUA-Net on quad-modal inputs, with a DSC of 0.821 and 95% HD of 3.21 mm, reflects the synergistic effect of its three core modules. The CMAM dynamically corrects inter-modal non-rigid deformations at the feature level, eliminating interference from residual errors introduced by traditional image-level registration and ensuring accurate cross-modal feature fusion. The MS-RDB, with its multi-scale receptive field design, enhances the network’s ability to capture weak boundary signals and contextual information of small lesions, laying a foundation for precise segmentation of tiny and diffuse lesions. Meanwhile, the MRT strategy, combining random modality masking and uncertainty-guided loss, significantly improves the model’s adaptability to arbitrary modality combinations, which is critical for its deployment in real clinical settings where modality loss is common.

The strong modality robustness of CMUA-Net further validates its clinical practical value. Even with only T2w input, the model maintains a DSC of 0.713, which is far higher than that of mainstream baseline methods under the same single-modality condition. In dual-modal and tri-modal scenarios, CMUA-Net still delivers stable and accurate segmentation results, with the T2w+DTI_FA combination achieving a DSC of 0.772, close to the performance of quad-modal input. This robustness stems from the MRT strategy’s adaptive adjustment of feature weights based on input modality completeness, which mitigates performance degradation caused by missing modalities. Additionally, the high consistency between the automated lesion volume measurements of CMUA-Net and manual physician measurements (R^2^ = 0.998) confirms the objectivity and reliability of the model’s quantitative results. This eliminates the subjective bias of manual measurement, reduces the clinician’s workload, and provides a standardized quantitative basis for clinical diagnosis and prognosis assessment.

The multi-center adaptability and stable performance across different lesion sizes and injury types highlight the generalization capability of CMUA-Net. The model achieves nearly consistent segmentation accuracy across two independent medical centers, with a DSC difference of only 0.96%, demonstrating its ability to overcome the heterogeneity of imaging equipment and scanning parameters across institutions. This feature makes it suitable for cross-institutional promotion and multi-center clinical research. For small lesions (< 50 mm^3^), CMUA-Net obtains a DSC of 0.756, outperforming all baseline methods, which is attributed to the MS-RDB’s multi-scale feature fusion mechanism that captures subtle lesion details. For traumatic and non-traumatic injuries, as well as lesions in cervical, thoracic, and lumbar spinal cord segments, CMUA-Net maintains high segmentation accuracy and boundary localization precision, reflecting its strong adaptability to the pathological and anatomical diversity of SCI lesions.

The 3D reconstruction performance of CMUA-Net provides important support for clinical surgical planning and doctor-patient communication. The low FVD of 18.7 indicates that the 3D model generated by the model is highly consistent with the real lesion morphology, accurately restoring the spatial relationship between the lesion and the surrounding spinal cord tissue. The key surgical parameters extracted from the 3D reconstruction results, such as the minimum lesion-spinal cord distance and the angle between the surgical path and the spinal cord axis, can guide surgeons to formulate individualized surgical plans, reduce intraoperative risks, and improve surgical safety. In addition, the intuitive 3D lesion model helps patients and their families visually understand the condition and surgical risks, enhancing communication efficiency and treatment compliance. Although this study has achieved satisfactory results, it still has limitations, such as the inclusion of only acute and subacute SCI patients and a relatively short follow-up period. Future research will expand the sample size to include chronic SCI patients, conduct long-term follow-up, and further optimize the model’s performance in extreme modality-loss scenarios, thereby better serving the full-cycle clinical management of SCI.

## CONCLUSION

The CMUA-Net proposed in this study effectively addressed core challenges in spinal cord injury lesion segmentation, including modal registration errors, modal gaps, and difficulties in modeling small lesions, in multimodal MRI data from 127 multicenter spinal cord injury patients. Through the synergistic effects of feature-level cross-modal alignment, multi-scale residual dense feature fusion, and modal-robust training strategies, this network significantly improved the objectivity and efficiency of spinal cord injury lesion analysis, providing reliable technical support for prognostic assessment, individualized surgical planning, and rehabilitation monitoring of spinal cord injuries. It possesses significant value for multicenter promotion and clinical application. However, this study also has limitations, including the inclusion of only acute and subacute phase patients and a relatively short follow-up period. Future studies will include chronic-phase cases and conduct long-term follow-up to further optimize the model's robustness in extreme modal gap scenarios. Furthermore, the practical application value of 3D reconstruction will be validated through clinical surgical subjective utility evaluation.

## Figures and Tables

**Fig. (1):**
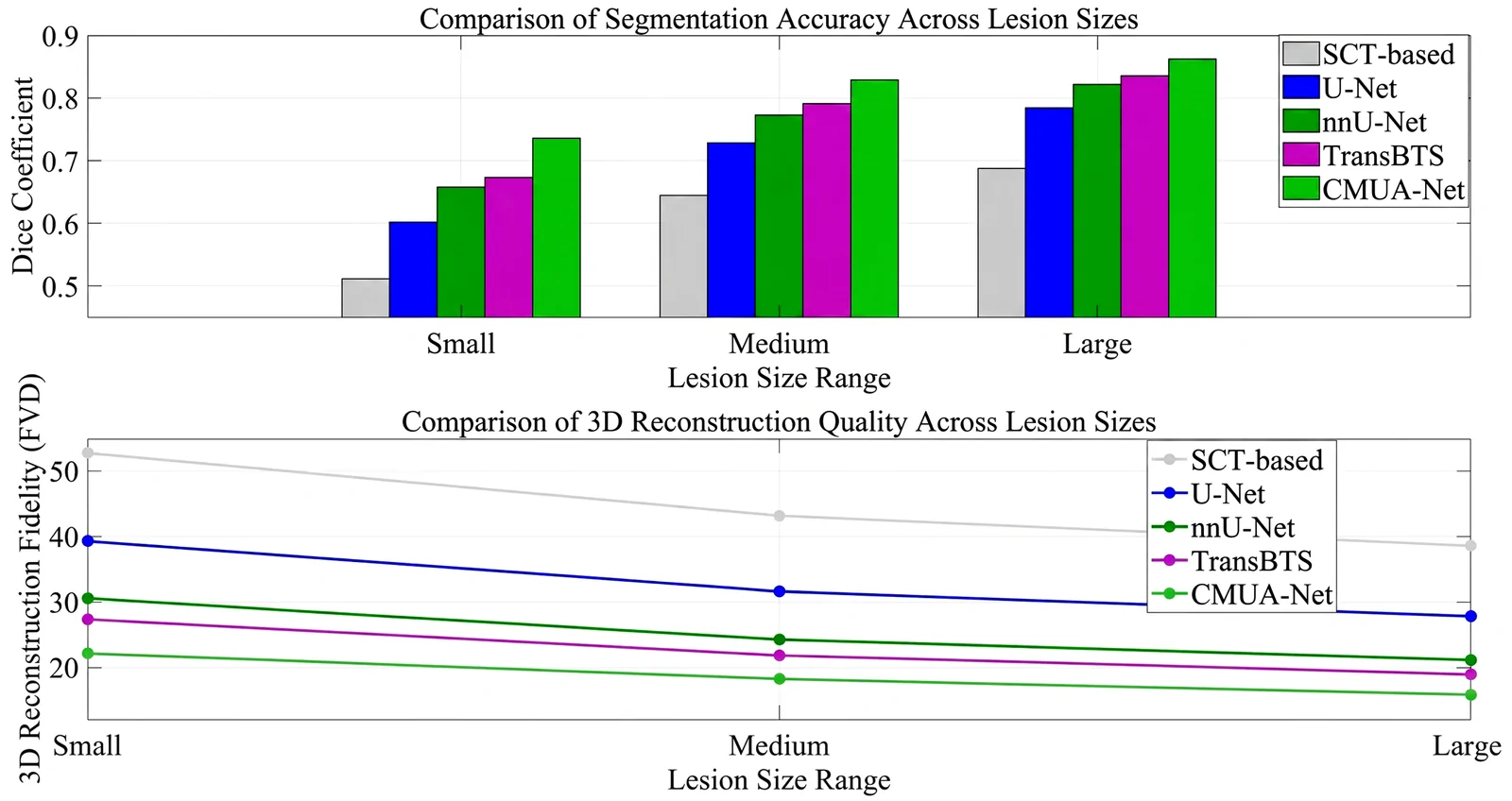
Performance differences of various methods in different lesion size ranges.

**Fig. (2):**
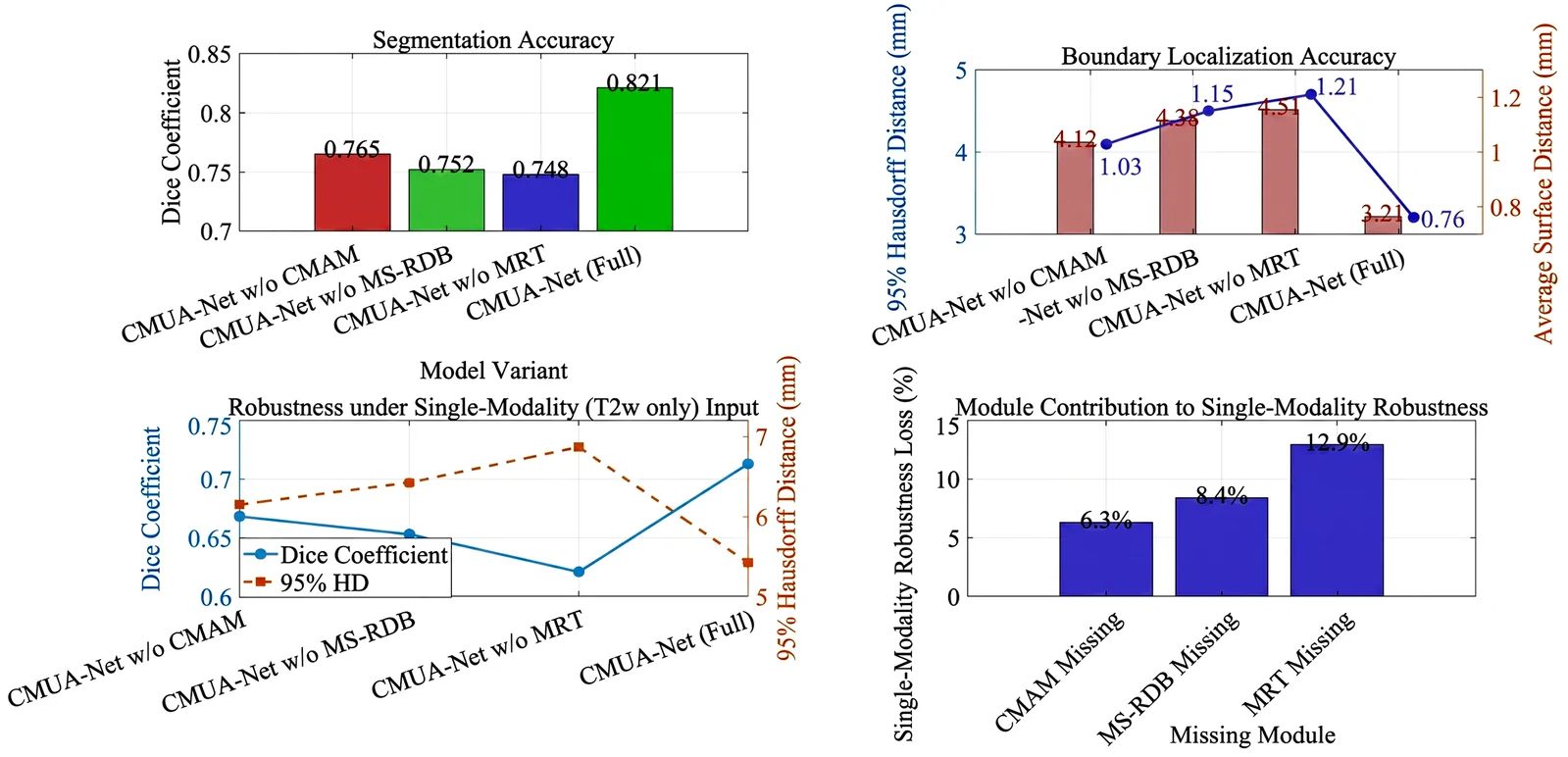
Impact of key modules on performance and robustness.

**Fig. (3):**
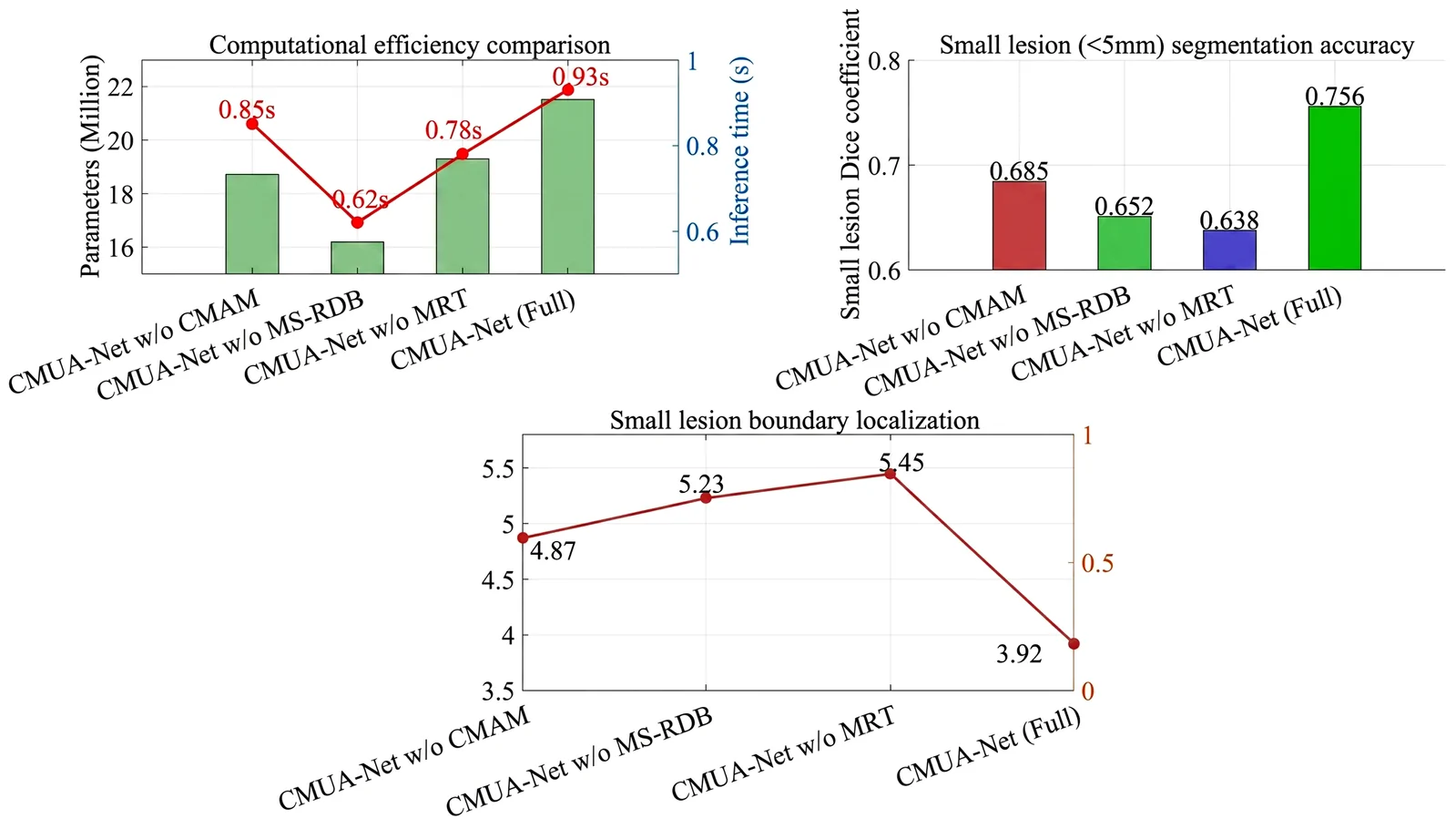
Impact of key modules on computational efficiency and microlesion segmentation.

**Fig. (4):**
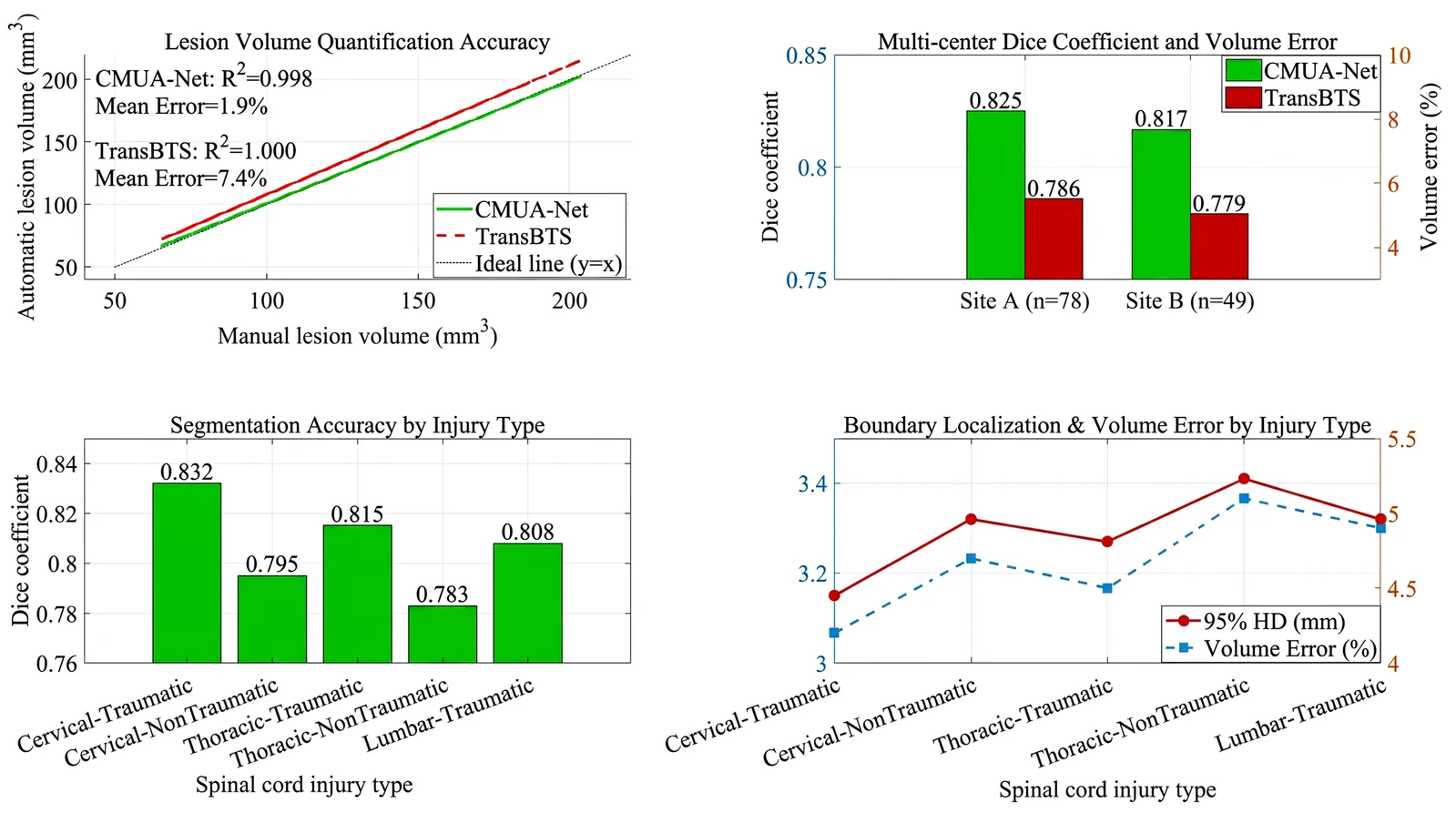
Lesion quantification accuracy and multi-center compatibility verification.

**Fig. (5):**
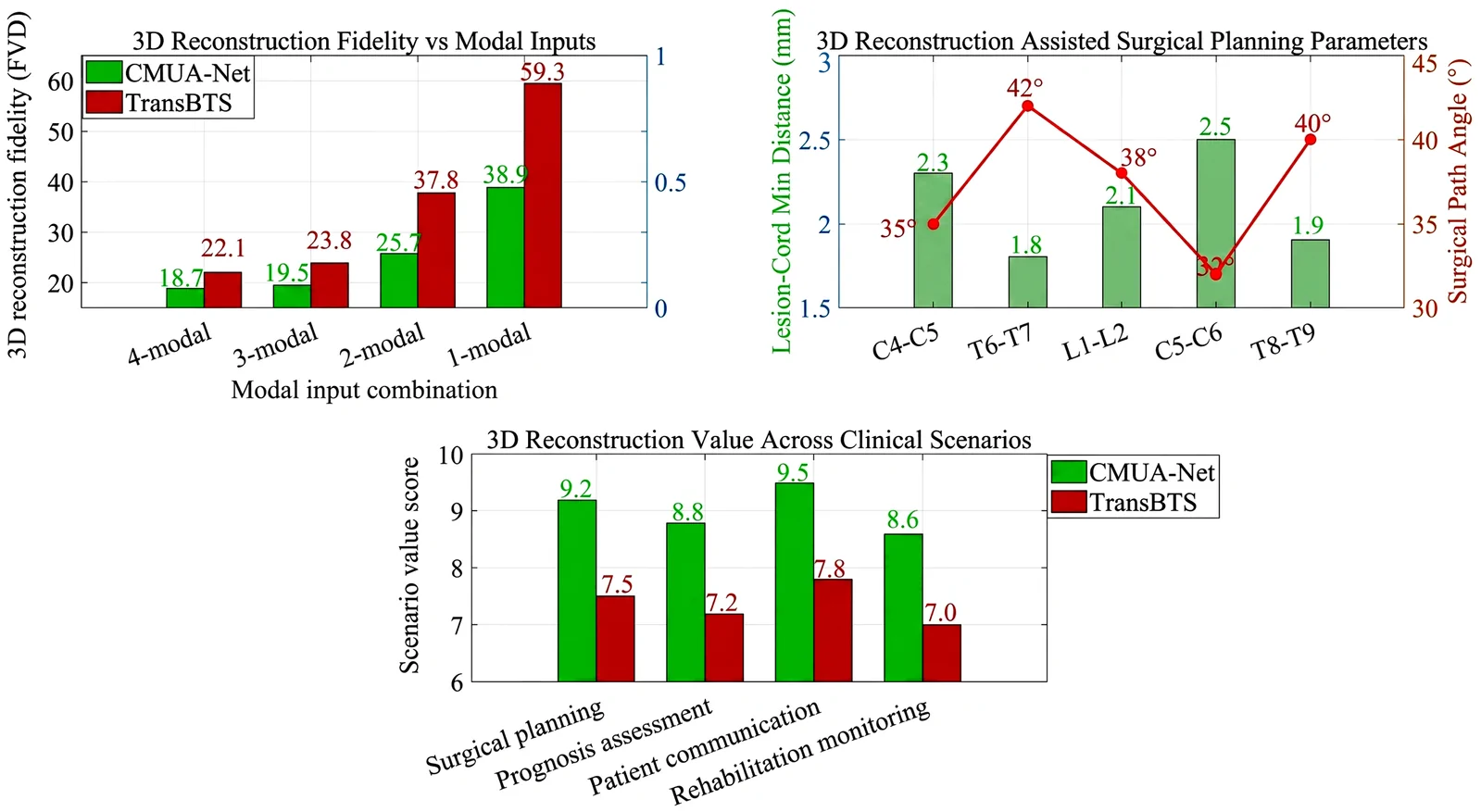
Verification of the clinical application value of CMUA-Net.

**Table 1:** Patient's basic information.

**Item**	**Site A(n = 78)**	**Site B(n = 49)**	**Total(N = 127)**
Gender (male/female)	52 / 26	33 / 16	85 / 42
Age (years)	38.2 ± 12.4	41.5 ± 13.1	39.5 ± 12.7
Injured segment distribution			
Cervical spinal cord (C1–C7)	51(65.4%)	30(61.2%)	81(63.8%)
Thoracic spinal cord (T1–T12)	22(28.2%)	16(32.7%)	38(29.9%)
Lumbar spinal cord (L1–L2)	5(6.4%)	3(6.1%)	8(6.3%)
Injury type			
Traumatic	69(88.5%)	44(89.8%)	113(89.0%)
Non-traumatic	9(11.5%)	5(10.2%)	14(11.0%)

**Table 2:** Comparison of segmentation performance of various methods under multimodal input.

**Evaluation Metrics**	**SCT-based Pipeline**	**U-Net**	**nnU-Net**	**TransBTS**	**CMUA-Net**
Dice similarity coefficient	0.632±0.087	0.715±0.072	0.768±0.061	0.783±0.058	0.821±0.053
95% Hausdorff distance (mm)	6.89±1.24	5.43±1.08	4.12±0.95	3.87±0.91	3.21±0.87
Mean surface distance (mm)	1.87±0.42	1.45±0.36	1.02±0.28	0.93±0.25	0.76±0.21
3D reconstruction fidelity (FVD)	45.3±6.2	32.7±5.1	25.4±4.3	22.1±3.8	18.7±3.2

**Table 3:** Key performance indicators of each method under a single modality.

**Modality Missing Solution**	**Evaluation Metrics**	**U-Net**	**nnU-Net**	**TransBTS**	**CMUA-Net**
T1w only	Dice	0.512±0.101	0.557±0.096	0.578±0.093	0.643±0.088
	95% HD(mm)	8.75±1.58	8.03±1.49	7.65±1.42	6.38±1.31
T2w only	Dice	0.582±0.093	0.625±0.087	0.641±0.082	0.713±0.075
	95% HD(mm)	7.92±1.45	7.15±1.32	6.83±1.27	5.42±1.13
DTI_FA only	Dice	0.495±0.102	0.531±0.098	0.557±0.095	0.628±0.089
	95% HD(mm)	9.23±1.61	8.57±1.53	8.12±1.48	6.95±1.36
T2*w only	Dice	0.503±0.100	0.542±0.097	0.563±0.094	0.635±0.087
	95% HD(mm)	9.01±1.59	8.35±1.51	7.98±1.45	6.72±1.33

**Table 4:** Key performance indicators of each method under dual-modality and tri-modality.

**Modality Missing Solution**	**Evaluation Metrics**	**U-Net**	**nnU-Net**	**TransBTS**	**CMUA-Net**
T1w+T2w	Dice	0.635±0.088	0.678±0.081	0.695±0.076	0.756±0.068
	95% HD(mm)	6.98±1.31	6.21±1.23	5.92±1.18	4.57±1.02
T1w+DTI_FA	Dice	0.612±0.090	0.653±0.084	0.672±0.080	0.732±0.071
	95% HD(mm)	7.35±1.38	6.62±1.30	6.28±1.24	4.95±1.07
T2w+DTI_FA	Dice	0.651±0.085	0.692±0.079	0.713±0.074	0.772±0.065
	95% HD(mm)	6.73±1.28	5.98±1.19	5.65±1.12	4.23±0.98
T2w+T2*w	Dice	0.643±0.086	0.685±0.08	0.702±0.075	0.764±0.067
	95% HD(mm)	6.85±1.30	6.12±1.21	5.81±1.15	4.41±1.00
T1w+T2w+DTI_FA	Dice	0.687±0.081	0.723±0.075	0.742±0.071	0.795±0.062
	95% HD(mm)	6.15±1.22	5.43±1.15	5.12±1.08	3.89±0.95
T1w+T2w+T2*w	Dice	0.675±0.083	0.712±0.077	0.731±0.073	0.786±0.064
	95% HD(mm)	6.32±1.25	5.65±1.18	5.33±1.10	4.05±0.97

**Table 5:** Key outcome events and summary indicators at different follow-up time points in cohort studies.

**Follow-up Time Point**	**Indicator Category**	**Specific Event Classification**	**Effective Sample Size**	**Number of Cases** **(n)**	**Proportion** **(%)**	**Mean Change in Correlation** **(± SD)**
1 month	Neurological functional outcome	Improvement in the ASIA motor score	122	48	39.3	Score increased by 4.2 ± 2.1 points
		Stable ASIA motor score (± 1 point)	122	65	53.3	-
		ASIA motor score decreased	122	9	7.4	Score decreased by 2.3 ± 1.5 points
	Outcome of lesion volume	Reduction in lesion volume	122	51	41.8	Volume reduction of 18.5 ± 8.3 mm ^3^
		Stable lesion volume (variation < 5%)	122	61	50	-
		Increased lesion volume	122	10	8.2	Volume increase of 12.1 ± 5.7 mm ^3^
3 month	Neurological functional outcome	Improvement in the ASIA motor score	119	62	52.1	Score increased by 6.8 ± 3.0 points
		Stable ASIA motor score (± 1 point)	119	47	39.5	-
		ASIA motor score decreased	119	10	8.4	Rating decreased by 2.5 ± 1.3 points
	Outcome of lesion volume	Reduction in lesion volume	119	73	61.3	Volume reduction of 29.7 ± 10.5 mm ^3^
		Stable lesion volume (variation < 5%)	119	36	30.3	-
		Increased lesion volume	119	10	8.4	Volume increase of 13.2 ± 6.1 mm ^3^

**Table 6:** Ablation experiment results of key modules.

**Model Variants**	**Dice**	**95% HD(mm)**	**Average Surface Distance (mm)**	**FVD**	**Dice Coefficient for Small Lesions**
CMUA-Net	0.821±0.053	3.21±0.87	0.76±0.21	18.7±3.2	0.756±0.061
CMUA-Net w/o CMAM	0.774±0.059	4.12±0.93	0.98±0.24	24.3±3.7	0.702±0.065
CMUA-Net w/o MS-RDB	0.758±0.062	4.38±0.96	1.05±0.26	26.8±4.1	0.652±0.072
CMUA-Net w/o MRT	0.732±0.065	4.75±1.01	1.21±0.29	29.5±4.5	0.683±0.068

## Data Availability

Not applicable.

## LIST OF ABBREVIATION

SCI: Spinal Cord Injury
3D: Three-Dimensional
MRI: Magnetic Resonance Imaging
CMUA-Net: Cross-Modal Alignment and Uncertainty-Aware Network
CMAM: Cross-Modal Attention Alignment Module
T1w: T1-weighted
DTI-FA: Diffusion Tensor Imaging-Fractional Anisotropy
T2*w: T2-star-weighted
T2w: T2-weighted
MS-RDB: Multi-Scale Residual Dense Block
MRT: Modality-Robust Training
DSC: Dice Similarity Coefficient
HD: Hausdorff Distance
FVD: Fréchet Video Distance
SCT: Spinal Cord Toolbox
nnU-Net: No-new U-Net
TransBTS: Transformer-based Brain Tumor Segmentation
FSL: FMRIB Software Library
ISNCSCI: International Standards for Neurological Classification of Spinal Cord Injury
MICE: Multiple Imputation using Chain Equations
MC-Dropout: Monte Carlo Dropout
ASD: Average Surface Distance
ASIA: American Spinal Injury Association
CNN: Convolutional Neural Network
SyN: Symmetric Normalization
STIR: Short TI Inversion Recovery
GMIM: Adaptive and Hierarchical Masked Image Modeling
MAE: Masked Autoencoder
CVPR: Computer Vision and Pattern Recognition Conference
SD: Standard Deviation
